# Supplementary material for: A mid-infrared lab-on-a-chip for dynamic reaction monitoring
Source: Nat Commun. 2022 Aug 13;13:4753. doi: 10.1038/s41467-022-32417-7 (PMC9376098; doi:10.1038/s41467-022-32417-7)
Supplement: Supplementary file 1 — Supplementary Information [file 41467_2022_32417_MOESM1_ESM.pdf]

Supplementary Information: A mid-infrared lab-on-a-chip for dynamic reaction monitoring

Borislav Hinkov,[1,*] Florian Pilat,[1] Laurin Lux,[2] Patricia L. Souza,[1,3] Mauro David,[1] Andreas Schwaighofer,[2] Daniela Ristanić,[1] Benedikt Schwarz,[1] Hermann Detz,[1,4] Aaron M. Andrews,[1] Bernhard Lendl,[2] and Gottfried Strasser,[1]

[1]Institute of Solid Stated Electronics & Center for Micro- and Nanostructures, TU Wien, 1040 Vienna, Austria
[2]Institute of Chemical Technologies and Analytics, TU Wien, 1060 Vienna, Austria
[3]LabSem-CETUC, Pontifícia Universidade Católica do Rio de Janeiro, Rio de Janeiro, Brazil
[4]CEITEC, Brno University of Technology, Brno, Czech Republic

[*]borislav.hinkov@tuwien.ac.at, http://www.qcllab.at

A. Bovine Serum Albumin (BSA)

Bovine serum albumin (BSA, $\geq$ 98.0%, Sigma-Aldrich, Steinheim, Germany) is a water-soluble monomeric protein with a molecular mass of 66.4 kDa [1]. It is one of the most studied proteins and is frequently employed as model protein for biophysical studies such as investigations of thermal denaturation. In this context, BSA was shown to have an increased tendency for protein aggregation at elevated concentrations and temperatures, particularly at high concentration formulations [2–4]. This process has been extensively studied using different analytical techniques, such as differential scanning calorimetry [2], static and dynamic light scattering [5] and fluorescence spectroscopy [1]. However, particularly IR spectroscopy offers the unique property of providing structural information of dynamic processes over a wide concentration range [2–4]. In IR spectroscopy, protein measurements are often performed in deuterated water (D_2O, 99.9 atom % D, Sigma-Aldrich, Steinheim, Germany) in order to avoid the overlap of the HOH-bending band of water with the protein amide I band. When working with a deuterated solution, the amide I band shows a shift of 5 - 10 cm^{-1} to lower wavenumbers and is then referred to as the amide I′ band [6]. Under ambient conditions, BSA predominantly consists of an α-helical secondary structure that results in an IR spectrum with an amide I′ band maximum at 1651 cm^{-1} in deuterated solution. This structure is stable up to 40 °C. At higher temperatures, heat induced denaturation occurs and conformational changes are partly reversible until approximately 60 °C. At higher temperatures irreversible aggregation occurs [7]. The progression of this aggregation process is strongly affected by the heating time, protein concentration, pH and salt concentrations. Heat-induced denaturation is accompanied by the formation of intermolecular β-sheets with associated IR bands at 1615 cm^{-1} and 1685 cm^{-1} in deuterated solution.
Figure 1(a) shows the amide I′ band in the 1580 - 1700 cm^{-1} spectral window. As reported in the literature [7,8], we can observe different transitions from the secondary structure of BSA.

B. QCLD Device Design

The device fabrication was performed in the in-house state-of-the-art cleanroom facilities ZMNS (Center for micro and nanostructures) at TU Wien. Table 1 shows the QCLD waveguide structure (the detailed AR sequence can be found in [9]).

**Material**	x (%)	Thickness (nm)	Doping (cm^{-3})
InP (substrate)	-	350 μm	2-4 x 10^{17}
$In_xGa_{1-x}As$	53	500	5 x 10^{16}
**37 x cascaded AR**			
$In_xGa_{1-x}As$	53	300	5 x 10^{16}
$In_xAl_{1-x}As$	52	600 + 800	1 x 10^{17} + 2 x 10^{17}
$In_xGa_{1-x}As$	53	350 + 10	8 x 10^{18} + 1 x 10^{19}

Supplementary Table 1: **QCLD waveguide structure.** For more details see [9].

The implemented DFB grating in the upper cladding further stabilizes the emitted wavelength of the QCL and thus increases the sensitivity of a QCLD-based sensor [10]. This allows the device to address a wider concentration range (<100 μg ml^{-1} to >90 mg ml^{-1}) and distinguish it from laser power fluctuations and occasional mode-hops. For adequate optical output power of the QCLs, we implemented weakly coupled DFB gratings. Even though leading to broader spectral modes (~2 cm^{-1}), they are still suitable to address the broad absorption features of BSA in D_2O.

C. QCLD Device Characterization

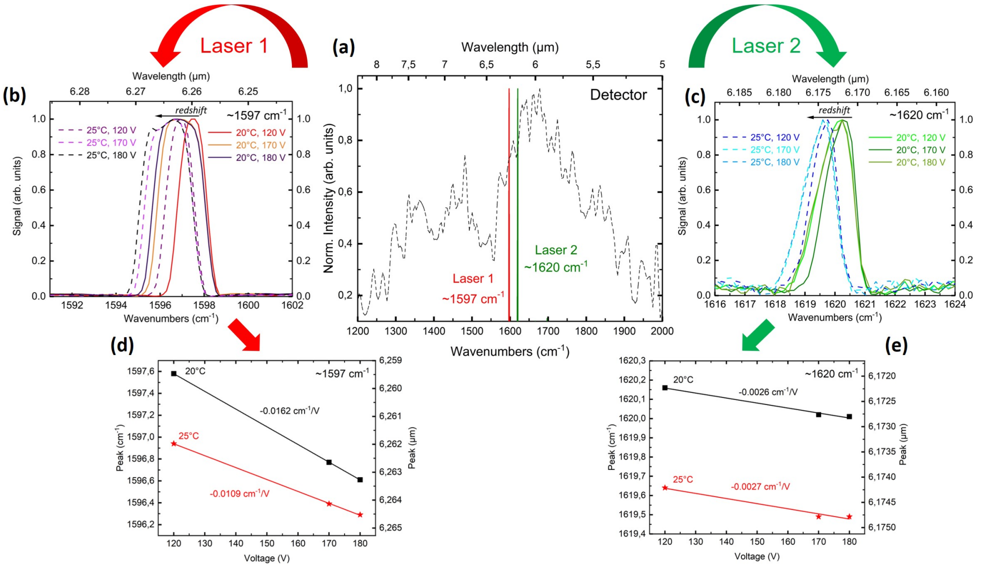

Supplementary Figure 1: **On-chip QCL and QCD characterization. a** Comparison of the QCD spectral response (black, dashed) with the spectral emission of Laser 1 around 1597 cm^{-1} (red) and Laser 2 around 1620 cm^{-1} (green). Laser tuning measurements at 1597 cm^{-1} are shown in **b** (spectra) and **d** (spectral peak tuning) and at 1620 cm^{-1} **c** (spectra), and **e** (spectral peak tuning)

Figure 1(a) shows the detailed analysis of the two lasers at ~1597 cm^{-1} and

$\sim$1620 cm^{-1} and their spectral overlap with the on-chip QCD. In addition, we analyzed the thermally/electrically induced spectral tuning under various laser driving conditions for pulsed operation (pulse length: 100 ns, repetition rate: 5 kHz) in Fig. 1(b) and (c) and observe continuous and mode-hop free tuning, as seen from Fig. 1(d) and (e).

D. Mid-IR Ellipsometry Measurements of PECVD SiN

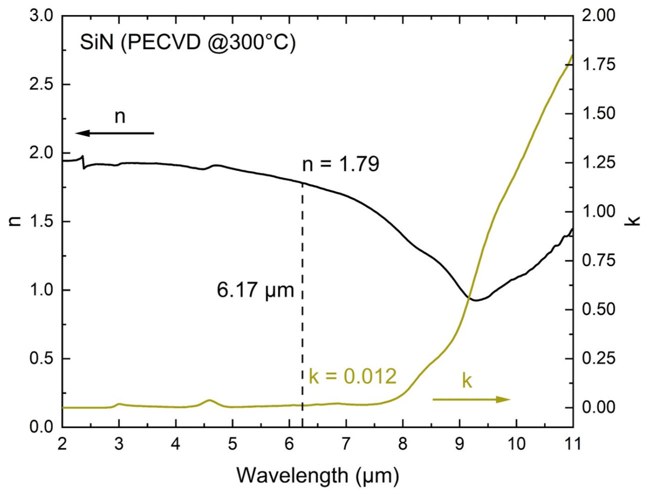

Supplementary Figure 2: **IR Ellipsometry data of PECVD-deposited SiN.** Optical constants of the SiN that was used as dielectric-loading layer in the on-chip DLSPP waveguide, measured between 2 μm and 11 μm wavelength.

We performed infrared ellipsometry measurements of SiN deposited with a regular PECVD (plasma enhanced chemical vapor deposition) reactor (Oxford Plasmalab 80 Plus) at 300 °C with the same recipe as for the DL layer of the on-chip DL-SPP waveguides. The values of n and k at the wavelength of interest of $\lambda \sim 6.17$ μm are extracted to be $n_{SiN} = 1.79$ and $k_{SiN} = 0.012$.

E. LOD Calculation

We use the standard definition for the LOD: LOD $= 3 \cdot$ std(t) slope^{-1} [11], where std(t) is the standard deviation of the measured signal during the signal recording time t and the slope is the slope m of the calibration function, as shown in Fig. 3 for the QCLD sensor.

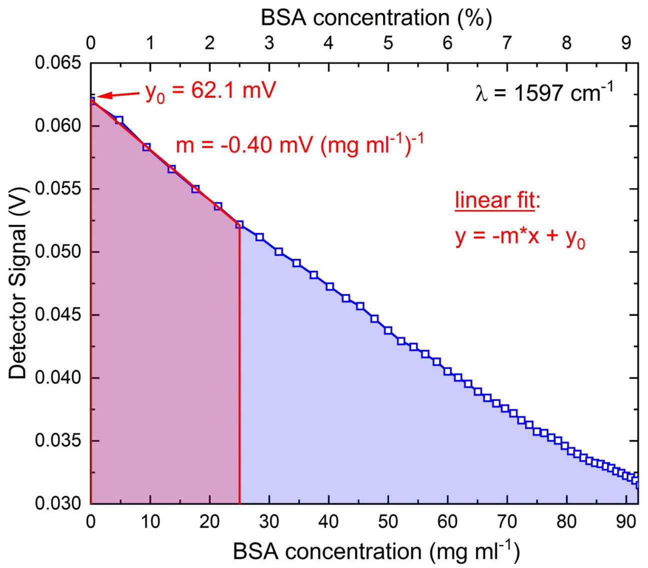

Supplementary Figure 3: **Concentration dependent detector signal measurement.** The detector signal (in V) is shown as function of the BSA concentration. We use a linear fit in the low concentration range up to 25 mg ml^{-1}, to fit the experimental data in the range of interest.

F. Heating Rate of BSA in the Thermal Denaturation Experiment

We compared the heating rates for the three BSA concentrations of 20, 40 and 60 mg ml^{-1} during the denaturation experiments. Figure 4 shows that the heating rates (expressed as their 1st derivative) are similar for all three concentrations.

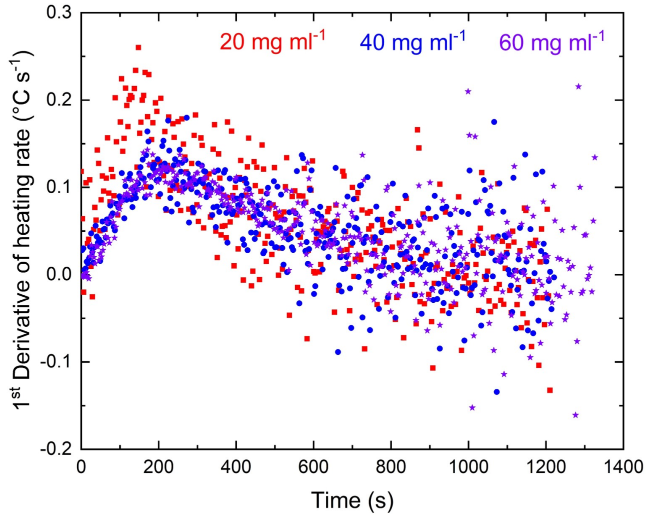

Supplementary Figure 4: **Heating rates per BSA concentration.** Comparison of the 1st derivative of the heating rate in (°C s^{-1}) for the three different BSA concentrations: 20 mg ml^{-1} (red), 40 mg ml^{-1} (blue) and 60 mg ml^{-1} (violet).

References

[1] V. A. Borzova, K. A. Markossian, N. A. Chebotareva, S. Y. Kleymenov, N. B. Poliansky, K. O. Muranov, V. A. Stein-Margolina, V. V. Shubin, D. I. Markov, and B. I. Kurganov, "Kinetics of thermal denaturation andaggregation of bovine serum albumin," PLoS One **11**, 1–29 (2016).

[2] S. Matheus, W. Friess, and H. C. Mahler, "FTIR and nDSC as analytical tools for high-concentration protein formulations," Pharm. Res. **23**, 1350–1363 (2006).

[3] D. Barreca, G. Laganà, S. Ficarra, E. Tellone, U. Leuzzi, S. Magazù, A. Galtieri, and E. Bellocco, "Anti-aggregation properties of trehalose on heat-induced secondary structure and conformation changes of bovine serum albumin," Biophys. Chem. **147**, 146–152 (2010).

[4] V. Militello, C. Casarino, A. Emanuele, A. Giostra, F. Pullara, and M. Leone, "Aggregation kinetics of bovine serum albumin studied by FTIR spectroscopy and light scattering," Biophys. Chem. **107**, 175–187 (2004).

[5] M. Panzica, A. Emanuele, and L. Cordone, "Thermal aggregation of bovine serum albumin in trehalose and sucrose aqueous solutions," J. Phys. Chem. B **116**, 11829–11836 (2012).

[6] H. Fabian and W. Mäntele, "Infrared Spectroscopy of Proteins," B. Handb. Vib. Spectrosc. (2006).

[7] K. Murayama and M. Tomida, "Heat-induced secondary structure and conformation change of bovine serum albumin investigated by Fourier transform infrared spectroscopy," Biochemistry **43**, 11526–11532 (2004).

[8] A. Schwaighofer, M. R. Alcaráz, C. Araman, H. Goicoechea, and B. Lendl, "External cavity-quantum cascade laser infrared spectroscopy for secondary structure analysis of proteins at low concentrations," Sci. Rep. **6**, 33556 (2016).

[9] B. Schwarz, P. Reininger, D. Ristanić, H. Detz, A. M. Andrews, W. Schrenk, and G. Strasser, "Monolithically integrated mid-infrared lab-on-a-chip using plasmonics and quantum cascade structures." Nat. Commun. **5**, 4085 (2014).

[10] D. Ristanic, B. Schwarz, P. Reininger, H. Detz, T. Zederbauer, A. M. Andrews, W. Schrenk, and G. Strasser, "Monolithically integrated mid-infrared sensor using narrow mode operation and temperature feedback," Appl. Phys. Lett. **106**, 041101 (2015).

[11] S. Freitag, M. Baer, L. Buntzoll, G. Ramer, A. Schwaighofer, B. Schmauss, and B. Lendl, "Polarimetric Balanced Detection: Background-Free Mid-IR Evanescent Field Laser Spectroscopy for Low-Noise, Long-term Stable Chemical Sensing," ACS Sensors **6**, 35–42 (2021).
