# Supplementary material for: A mid-infrared lab-on-a-chip for dynamic reaction monitoring
Source: Nat Commun. 2022 Aug 13;13:4753. doi: 10.1038/s41467-022-32417-7 (PMC9376098; doi:10.1038/s41467-022-32417-7)
Supplement: Supplementary file 3 — Reporting Summary [file 41467_2022_32417_MOESM3_ESM.pdf]

nature portfolio

Corresponding author(s): Borislav Hinkov

Last updated by author(s): Jul 13, 2022

Reporting Summary

Nature Portfolio wishes to improve the reproducibility of the work that we publish. This form provides structure for consistency and transparency in reporting. For further information on Nature Portfolio policies, see our Editorial Policies and the Editorial Policy Checklist.

Statistics

For all statistical analyses, confirm that the following items are present in the figure legend, table legend, main text, or Methods section.

n/a	Confirmed	
x		The exact sample size (n) for each experimental group/condition, given as a discrete number and unit of measurement
	x	A statement on whether measurements were taken from distinct samples or whether the same sample was measured repeatedly
	x	The statistical test(s) used AND whether they are one- or two-sided<br>*Only common tests should be described solely by name; describe more complex techniques in the Methods section.*
x		A description of all covariates tested
x		A description of any assumptions or corrections, such as tests of normality and adjustment for multiple comparisons
	x	A full description of the statistical parameters including central tendency (e.g. means) or other basic estimates (e.g. regression coefficient) AND variation (e.g. standard deviation) or associated estimates of uncertainty (e.g. confidence intervals)
x		For null hypothesis testing, the test statistic (e.g. F, t, r) with confidence intervals, effect sizes, degrees of freedom and P value noted<br>*Give P values as exact values whenever suitable.*
x		For Bayesian analysis, information on the choice of priors and Markov chain Monte Carlo settings
x		For hierarchical and complex designs, identification of the appropriate level for tests and full reporting of outcomes
x		Estimates of effect sizes (e.g. Cohen's d, Pearson's r), indicating how they were calculated

Our web collection on statistics for biologists contains articles on many of the points above.

Software and code

Policy information about availability of computer code

Data collection	Python 3.9
Data analysis	OPUS 8.1, Origin 9.8, COMSOL Multiphysics 5.5, Autodesk Fusion 360 V2.0.11894

For manuscripts utilizing custom algorithms or software that are central to the research but not yet described in published literature, software must be made available to editors and reviewers. We strongly encourage code deposition in a community repository (e.g. GitHub). See the Nature Portfolio guidelines for submitting code & software for further information.

Data

Policy information about availability of data

All manuscripts must include a data availability statement. This statement should provide the following information, where applicable:

- Accession codes, unique identifiers, or web links for publicly available datasets
- A description of any restrictions on data availability
- For clinical datasets or third party data, please ensure that the statement adheres to our policy

The data generated in this study are provided in the Supplementary Source Data file.

Field-specific reporting

Life sciences study design

All studies must disclose on these points even when the disclosure is negative.

Sample size	In the proof-of-concept study, more than 5 individual devices were characterized in the course of the experiments. In total more than 20 individual BSA in D2O samples were produced at various concentrations and either directly used for measurements or used as stock solutions and diluted. Sample size during the experiments was defined through averaging the sampled data, typically for 2 seconds per displayed measurement point (calibration line measurements, denaturation experiments)
Data exclusions	No data excluded from data analysis.
Replication	For the performed proof-of-concept measurements, reproducibility was confirmed by replication of the reference measurements in the D2O matrix four times. The recorded data for the quantitative measurements, follows the expected Beer-Lamberts Law and the results of the denaturation study agree with published reference studies, confirming the validity of the measurements.
Randomization	Randomization was not relevant to our study, since we show a proof-of-concept measurement of a device (no "participants", "organisms", etc. involved), where we do not expect relevant hidden variables ("lurking variables", e.g. unknown pre-conditions in clinical studies). This is validated by the results that agree with published reference studies , confirming the validity of our measurements and study design.
Blinding	Opposed to e.g. clinical research studies, where blinding is carried out as part of randomized controlled trials with a control (Placebo) group, blinding was not relevant to our proof-of-concept study of a device. We do not expect a relevant level of biased interpretation of the results, since the measurements were performed with standard lab equipment using standardized data evaluation routines (e.g. reference measurement, sample measurement, calculation of absorbance, plot and compare) and are much less subject to interpretation (where bias would play an important role), as compared to e.g. clinical trials.

Reporting for specific materials, systems and methods

We require information from authors about some types of materials, experimental systems and methods used in many studies. Here, indicate whether each material, system or method listed is relevant to your study. If you are not sure if a list item applies to your research, read the appropriate section before selecting a response.

Materials & experimental systems

n/a	Involved in the study	
☒	☐	Antibodies
☒	☐	Eukaryotic cell lines
☒	☐	Palaeontology and archaeology
☒	☐	Animals and other organisms
☒	☐	Human research participants
☒	☐	Clinical data
☒	☐	Dual use research of concern

Methods

n/a	Involved in the study	
☒	☐	ChIP-seq
☒	☐	Flow cytometry
☒	☐	MRI-based neuroimaging
